# Multidrug-Resistant Enteropathogenic *Escherichia coli* Isolated from Diarrhoeic Calves, Milk, and Workers in Dairy Farms: A Potential Public Health Risk

**DOI:** 10.3390/antibiotics11080999

**Published:** 2022-07-25

**Authors:** Ibrahim E. Eldesoukey, Walid Elmonir, Abdulaziz Alouffi, Eman I. M. Beleta, Mohamed A. Kelany, Shimaa Samir Elnahriry, Mohammed Ibrahim Alghonaim, Zeyad Abdullah alZeyadi, Haitham Elaadli

**Affiliations:** 1 Department of Bacteriology, Mycology and Immunology, Faculty of Veterinary Medicine, Kafrelsheikh University, Kafrelsheikh 33516, Egypt; 2 Department of Hygiene and Preventive Medicine (Zoonoses), Faculty of Veterinary Medicine, Kafrelsheikh University, Kafrelsheikh 33516, Egypt; 3 King Abdulaziz City for Science and Technology, Riyadh 12354, Saudi Arabia; asn1950r@gmail.com; 4 Department of Brucellosis Research, Animal Health Research Institute, Agricultural Research Center, Giza 12618, Egypt; emanasser.eb@gmail.com; 5 Department of Microbiology, The Central Laboratory of Residue Analysis of Pesticides and Heavy Metals in Food, Agricultural Research Center, Giza 12311, Egypt; mohamed_kelany@hotmail.com; 6 Department of Bacteriology, Mycology and Immunology, Faculty of Veterinary Medicine, University of Sadat City, Sadat City 32897, Egypt; kamelsamir95@yahoo.com; 7 Department of Biology, College of Science, Imam Muhammad bin Saud Islamic University, Riyadh 11623, Saudi Arabia; mialghonaim@imamu.edu.sa; 8 Department of Clinical Laboratory Sciences, College of Applied Medical Sciences, Shaqra University, Dawadmi 11961, Saudi Arabia; zalzeyadi@su.edu.sa; 9 Department of Animal Hygiene and Zoonoses, Faculty of Veterinary Medicine, Alexandria University, Alexandria 22758, Egypt; haytham.kamal@alexu.edu.eg

**Keywords:** EPEC, antimicrobial resistance, diarrhoeic calves, milk, worker, Egypt

## Abstract

Enteropathogenic *Escherichia coli* (EPEC) is a leading cause of diarrhoeagenic diseases in humans and cattle worldwide. The emergence of multidrug-resistant (MDR) EPEC from cattle sources is a public health concern. A total of 240 samples (75 diarrhoeic calves, 150 milk samples, and 15 workers) were examined for prevalence of EPEC in three dairy farms in Egypt. Antimicrobial resistance (AMR) traits were determined by antibiogram and polymerase chain reaction (PCR) detection of β-lactamase-encoding genes, plasmid-mediated quinolone resistance genes, and carbapenemase-encoding genes. The genetic relatedness of the isolates was assessed using repetitive extragenic palindromic sequence-based PCR (REP-PCR). EPEC isolates were detected in 22.7% (17/75) of diarrhoeic calves, 5.3% (8/150) of milk samples, and 20% (3/15) of worker samples. The detected serovars were O26 (5%), O111 (3.3%), O124 (1.6%), O126 (0.8%), and O55 (0.8%). AMR-EPEC (harbouring any AMR gene) was detected in 9.2% of samples. Among isolates, *bla*_TEM_ was the most detected gene (39.3%), followed by *bla*_SHV_ (32.1%) and *bla*_CTX-M-1_ (25%). The *qnrA*, *qnrB*, and *qnrS* genes were detected in 21.4%, 10.7%, and 7.1% of isolates, respectively. The *bla*_VIM_ gene was detected in 14.3% of isolates. All EPEC (100%) isolates were MDR. High resistance rates were reported for ampicillin (100%), tetracycline (89.3%), cefazolin (71%), and ciprofloxacin (64.3%). Three O26 isolates and two O111 isolates showed the highest multiple-antibiotic resistance (MAR) indices (0.85–0.92); these isolates harboured *bla*_SHV-12_ and *bla*_CTX-M-15_ genes, respectively. REP-PCR genotyping showed high genetic diversity of EPEC, although isolates belonging to the same serotype or farm were clustered together. Two worker isolates (O111 and O26) showed high genetic similarity (80–95%) with diarrhoeic calf isolates of matched serotypes/farms. This may highlight potential inter-species transmission within the farm. This study highlights the potential high risk of cattle (especially diarrhoeic calves) as disseminators of MDR-EPEC and/or their AMR genes in the study area. Prohibition of non-prescribed use of antibiotics in dairy farms in Egypt is strongly warranted.

## 1. Introduction

Enteropathogenic *Escherichia coli* (EPEC) strains are leading etiological agents of diarrhoeagenic diseases in infants [1]. EPEC isolates have also been implicated in several diarrhoeic outbreaks among adults and AIDS patients worldwide [2,3]. In cattle, EPEC has been isolated from diarrhoeic calves and healthy carriers in several countries [4,5,6]. The EPEC pathotype is defined by the acquisition of an attaching and effacing (*eae*) gene with a lack of Shiga toxin-producing (*stx1* and *stx2*) genes [1]. The *eae* gene encodes intimin, an outer-membrane protein responsible for attaching bacteria to the intestinal epithelium and effacing (destroying) the intestinal villi (A/E lesion). EPEC isolates that lack the EPEC adherence factor (EAF) plasmid that encodes the bundle-forming pilus (BFP) are atypical EPEC (aEPEC) [3]. aEPEC has mostly been isolated from animals, although has recently emerged as a human diarrhoeagenic pathogen [2,3]. 

Antimicrobial resistance among animal pathogens has recently emerged as a major threat to public health [7,8]. *E. coli* (including EPEC) isolates are important inter- and intraspecies disseminators of antimicrobial resistance (AMR) genes [9,10,11].

Cattle and their milk are important reservoirs of *E. coli*-carrying AMR genes [11,12,13]; multidrug resistance (MDR) selection in this host is driven by the excess, non-prescribed use of antibiotics for the prevention or treatment of bacterial infections of economic importance, such as neonatal calf diarrhoea [11,12]. MDR *E. coli* carried by cattle can pass to humans through contact with live carrier animals or through consumption of their food products [14,15].

The extended-spectrum β-lactamase (ESBL)-producing genes, plasmid-mediated quinolone resistance (PMQR) genes, and carbapenemase-producing (CR) genes are leading contributors to resistance against β-lactam, fluoroquinolone, and carbapenem antibiotics, respectively. β-lactams and fluoroquinolones are the most commonly used antibiotics in clinical human medicine and veterinary practices worldwide [7]. Carbapenems are among the last-resort antibiotics for the treatment of MDR pathogens in humans. This class of antibiotics is prohibited for treatment of animals in several countries, yet there are several reports of emerging resistance against these antibiotics in isolates from cattle sources [13,16], posing a major public health threat. 

In Egypt, the role of dairy cattle in the spread of EPEC and the prevalence of antimicrobial resistance in isolates of this pathotype is largely unknown. Therefore, the aim of this study aims are (1) to investigate the distribution frequency of EPEC isolates from diarrhoeic calves, milk, and workers in dairy farms in Egypt, (2) to record the antibiogram of isolates and their associated AMR genetic determinants, and (3) to assess the genetic relatedness between isolates using repetitive extragenic palindromic sequence-based PCR (REP-PCR) for evidence of potential infection pathways within farms. 

## 2. Results

In this study, we isolated 28 EPEC isolates (28/240, 11.7%) from diarrhoeic calves (17/75, 22.7%), milk samples (8/150, 5.3%), and workers (3/15, 20%) in three dairy farms (Table 1). The EPEC isolates were 5.2 and 4.4 times more likely to be associated with diarrhoeic calves and workers than milk, respectively (*p* ˂ 0.001; *p* < 0.04) (Table 1).

All detected EPEC isolates lacked the *bfpA* gene and were therefore defined as aEPEC. We detected five different serovars of aEPEC among examined samples (Table 2): O26 (5%), O111 (3.3%), O124 (1.7%), O126 (0.8%), and O55 (0.8%). O26 was the most detected serovar in diarrhoeic calves (7/75, 9.3%) and milk samples (4/150, 2.7%). The O126 and O55 serovars were only detected in diarrhoeic calves (Table 2). 

EPEC isolates that harboured at least one antimicrobial resistance gene (AMR-EPEC) were detected in 9.2% (22/240) of samples. The rates of AMR-EPEC isolates per source were 18.7% per diarrhoeic calves, 4% per milk sample, and 13.3% per worker sample (Table 1). These isolates were more likely to be associated with diarrhoeic calf samples (OR = 5.5, *p* = 0.001) than with milk samples (Table 1). ESBL-EPEC (harboured any ESBL genes), PMQR-EPEC (harboured any PMQR genes), and CR-EPEC (harboured any CR genes) were detected in 8.8%, 2.9%, and 1.7% of samples, respectively (Table 1). ESBL-EPEC and PMQR-EPEC were more likely to be associated with diarrhoeic calves compared to milk (OR = 5.03, *p* = 0.002; OR = 12.9, *p* = 0.02). The CR-EPEC isolates were 1.7 times more likely to be associated with dairy workers than diarrhoeic samples; however, this difference was not significant (*p* = 0.7). 

**Figure 1:**
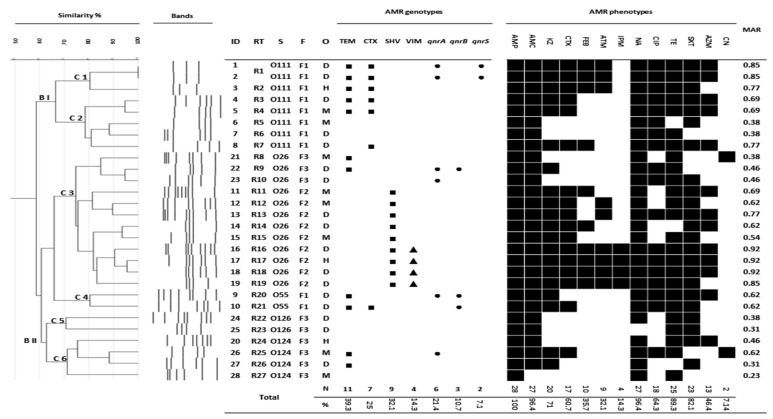
REP-PCR genotyping, virulence genes, and AMR of EPEC isolates recovered from diarrhoeic calves, milk, and workers in studied dairy farms. ■, ESBL genes; ▲, CR genes; ●, PMQR genes.

**Figure 2:**
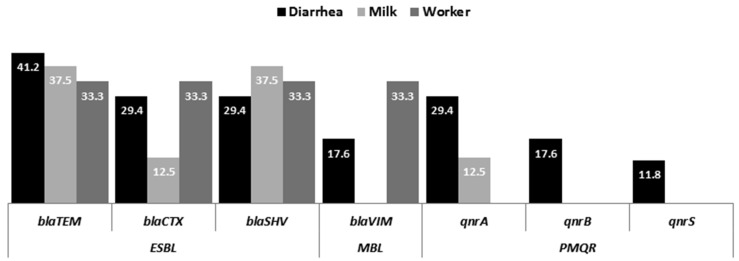
Frequency distribution of AMR genes in isolates recovered from diarrhoeic calves, milk, and workers in studied dairy farms.

Most ESBL and PMQR genes were predominant in isolates from diarrhoeic calves (Figure 1 and Figure 2). However, no significant difference was observed in the rate of AMR genes acquired between isolates from different sources (*p* > 0.05). CR genes were not found in isolates from milk, and PMQR genes were not detected in any worker samples (Figure 1 and Figure 2 and Table 3). 

The distribution of individual AMR genes ranged from 7.1% to 39.3% of isolates (Figure 1). *bla*_TEM_ was the most detected gene (39.3%), followed by *bla*_SHV_ (32.1%) and *bla*_CTX-M-1_ (25%). *qnrA* was the most prevalent PMQR gene detected in EPEC (21.4%). *bla*_VIM_ was detected in 14.3% of isolates. None of the isolates harboured *bla*_OXA-1_, *bla*_NDM-1_, or *bla*_IMP_ genes. 

We genotyped the isolates into four groups based on the detected class of AMR genes (Table 3): I, ESBL/- (alone) genotype (11 isolates, 39.3%); II, PMQR/- genotype (one isolate, 3.6%); III, ESBL/PMQR genotype (six isolates, 21.4%); and IV, ESBL/CR genotype (four isolates, 14.3%). *bla*_SHV_/-, *bla*_SHV_/*bla*_VIM_, and *bla*_TEM-1_/*bla*_CTX-M-1_ were the most detected genotypes, with a percentage of 17.9% (five isolates), 14.3% (four isolates), and 10.7% (three isolates), respectively (Table 3). Isolates with *bla*_SHV_/- (two diarrhoeic calves and three milk samples) and *bla*_SHV_/*bla*_VIM_ (three diarrhoeic calves and one worker) genotypes belonged to the O26 serovar, whereas isolates with the *bla*_TEM-1_/*bla*_CTX-M-1_ genotype (one diarrhoeic calf, one milk sample, and one worker) belonged to serovar O111 (Figure 1). Two O111 isolates (two diarrhoeic calves) carried four genes (*bla*_TEM_/*bla*_CTX-M-1_/*qnrA*/*qnrS*) (Figure 1). 

All 28 EPEC (100%) isolates were MDR to at least three classes of antibiotics, with an MAR index ranging from 0.23 to 0.92 (Figure 1). The highest resistance rates were reported for AMP (100%), AMC (96.4%), NA (96.4%), TE (89.3%), SXT (82.1%), and KZ (71%). Approximately two-thirds (64.3%) of the isolates were resistant to CIP. The lowest resistance rates were reported for CN (7.1%), IPM (14.3%), and ATM (32.1%). There was a concordance between the acquisition of ESBL, CR, and PMQR genes and the expression of phenotypic resistance to β-lactam, carbapenem, and fluoroquinolones in all studied EPEC isolates, respectively (Figure 1). Few isolates that lacked any of investigated AMR genes showed phenotypic resistance to β-lactam and/or fluoroquinolones (ID 6, 7, 20, 24, 25, and 28) (Figure 1). The O26 isolates carrying combined ESBL and CR genes (*bla*_SHV_/*bla*_VIM_) and the O111 isolates carrying combined ESBL and PMQR genes (*bla*_TEM_/*bla*_CTX-M-1_/*qnrA*/*qnrS*) showed the highest MAR indices (0.85–0.92). 

We sequenced the *bla*_CTX-M-1_ gene of two O111 isolates (ID 2 from diarrhoeic calves and ID 3 from a worker) and three *bla*_SHV_ genes of three isolates of O26 (ID 13 and 18 from diarrhoeic calves and ID 17 from a worker). Analysis of *bla*_CTX-M-1_ of ID 2 (MW721313) and ID 3 (MW721311) isolates showed 99.8% and 100% similarity, respectively, to *bla*_CTX-M-15_ of several *E. coli* isolates in the GenBank database (including CP081589, CP075059, CP059120, and CP048916) (Figure S1). The difference in the *bla*_CTX-M-1_ gene of ID 2 isolate is attributed to a single-nucleotide mutation (G to T at position 21); however, this point mutation did not alter the coded amino acid (valine) (Figure S2). The sequenced *bla*_SHV_ gene of the three isolates (ID 13, MW721318; ID 18, MW721319; and ID 17, MW721320) showed 100% similarity with *bla*_SHV-12_ from several *E. coli* isolates in the GenBank database (for example, MH460799, CP046002, and CP048293).

REP-PCR-based genotyping analysis of the 28 EPEC isolates is shown in Figure 1. The band patterns ranged from three to nine bands, with a size range of 150 to 2100 bp. The dendrogram map classified the EPEC isolates into two branches (BI and BII), six clades (C1–6), and 27 REP genotypes (RTs), with a discrimination index of 0.997. The EPEC isolates showed high genetic diversity, although isolates that belonged to the same serotype were clustered together. Unlike serotype, the source of the isolate did not discriminate between the isolates. The eight isolates of the O111 serovar belonging to the same farm (F1) displayed seven RTs with 80–95% genetic similarity. Six O111 isolates carried combined *bla*_TEM_/*bla*_CTX-M-1_ genes; two of these isolates (two diarrhoeic calves: ID 1 and 2) carried two additional PMQR genes (*bla*_TEM_/*bla*_CTX-M-1_/*qnrA*/*qnrS* genotype) and showed identical RT (R1; Figure 1). One worker isolate (ID 3) showed high genetic similarity (>80%) with two isolates from diarrhoeic calves (ID 1 and 2), and the three isolates shared the same clade (C1). Twelve O26 isolates belonging to C3 displayed eleven RTs (R8–19) with 75–95% similarity. Nine O26 isolates from the same farm (F2) contained *bla*_SHV_; of these, four isolates in one cluster (three diarrhoeic calves and one worker) carried an additional *bla*_VIM_ gene (*bla*_SHV_/*bla*_VIM_ genotype). 

## 3. Discussion

The overall prevalence of EPEC in this study was 11.7%. The highest rate of EPEC was detected in diarrhoeic calves (22.7%). Lower prevalence rates (4.5–9.7%) were reported in Egypt [6], India [4], and Turkey [5]; however, EPEC was recovered at a higher rate (38%) from diarrhoeic calves in Belgium [17]. In contrast, EPEC was not a common cause of calf diarrhoea in dairy farms in Sweden [12]. In this study, EPEC was recovered from 5.3% of milk samples from healthy dairy cattle. In agreement with these results, low rates of EPEC (0.9–4.5%) were reported in milk worldwide [18,19,20]; however, dairy cattle were reported to be faecal carriers with relatively high rates (31–36%) of EPEC [21,22]. In addition, Lambertini et al. [21] suggested that milk seems play a smaller role than played by cattle faeces in environmental and interspecies dissemination of pathogenic *E. coli* in dairy farms. In support of this suggestion, our findings showed that EPEC isolates were 5.5 times more likely to be associated with diarrhoeic calves than with milk (OR = 5.5, *p* = 0.001). In this study, EPEC was detected in 20% of workers’ stool samples. In agreement with these results, dairy farm workers were previously reported as carriers of pathogenic *E. coli* [14,23]. This may highlight the critical role of farm workers in infection dynamics within dairy farms. 

In this investigation, all EPEC isolates were atypical (aEPEC; lacking the *bfp* gene), which is in agreement with another report from China [20]. Four of the five detected aEPEC serovars (O26, O111, O126, and O55) belonged to the classical EPEC (typical and atypical) serovars defined by the World Health Organization [1]. O124, a non-classical aEPEC serovar, was detected in 1.7% of samples. Similarly, non-classical aEPEC serovars were detected worldwide, especially from cattle sources [3]. O26 was the predominant serovar (detected in 5% of samples and 42.9% of EPEC isolates), which is in line with several reports worldwide [4,17]. The O26, O111, and O55 serovars were associated with several outbreaks of aEPEC diarrhoea cases among children and adults worldwide [1,2,3], which highlights the public health risk of these serovars.

AMR-EPEC isolates with at least one antimicrobial resistance gene were detected in 11.7% of samples. These isolates were more likely to be associated with diarrhoeic calves than with milk samples from healthy cattle (18.7% vs. 4%; OR = 5.5, *p* = 0.001). Similarly, ESBL-EPEC (17.3% vs. 4%) and PMQR-EPEC (8% vs. 0.7%) were more likely to be associated with diarrhoeic calves than with milk (*p* = 0.002–0.02). These findings are consistent with those reported by de Verdier et al. [12], suggesting a greater association of antibiotic resistance with clinical diarrhoeic calves than healthy calves. Overuse and misuse of antibiotics for prophylaxis or therapy in young calves may have contributed to our findings, as previously described [11,12]. Additionally, the development of resistance among *E. coli* isolates from calves may be attributed to drinking milk containing antibiotic residues from treated cows on the same farm [24].

ESPL-EPEC isolates were detected in 13.3% of samples from dairy farm workers. This result is comparable with that of another report (12.5%) from Germany [14]. Unlike diarrhoeic calf samples, there was no difference between worker and milk samples in terms of the prevalence of ESPL-EPEC isolates (*p* = 0.1). This suggest that diarrhoeic cases may contribute more to the dissemination of ESPL-EPEC within the farm compared to other sources. In contrast, workers may contribute more than or equally to diarrhoeic calves in the spread of CR-EPEC, highlighting their important role as a source of these pathogens (OR = 1.7, *p* = 0.7). 

In this study, the frequency of individual AMR genes ranged from 7.1% to 39.3% among isolates from different sources. This is in agreement with other reports (10.4–34.3%) in Egypt and elsewhere [25]. 

The *bla*_TEM_, *bla*_SHV_, and *bla*_CTX-M-1_ ESBL genes were detected at high rates (25–39.3%) among EPEC isolates from various sources. Similarly, these ESBL genes were detected at variable rates in cattle and workers on dairy farms worldwide [10,14,25]. None of the detected isolates in this study harboured *bla*_OXA1_; this gene is usually detected at low rates (1–3%) in *E. coli* isolates from dairy farms in Egypt [13,25]. 

In this study, sequence analysis showed that the *bla*_CTX-M-15_ and *bla*_SHV-12_ genes were detected in O111 isolates (one diarrhoeic calf and one worker) and O26 isolates (two diarrhoeic calves and one worker), respectively. In agreement with these results, Dandachi et al. [11] reported that *bla*_CTX-M-1_ (including *bla*_CTX-M-15_) and *bla*_SHV-12_ are the most prevalent ESBL genes in isolates from animals in the Mediterranean Basin (including Egypt). MDR *E. coli* isolates that harbour *bla*_CTX-M-15_ and/or *bla*_SHV-12_ genes have been associated with several clinical outbreaks among humans and cattle worldwide [10,11,25]. Additionally, both EPEC O111 and O26 isolates were found to carry multiple AMR genes, and they showed the highest MAR indices (O111 also carried *bla*_TEM1_ and PMQR genes; O26 also carried *bla*_VIM_). This highlights the emerging risk of these isolates with respect to both cattle and public health in the study area. 

In the current study, PMQR genes were detected in diarrhoeic calves and milk isolates (12.5–35.3%). A comparable rate (36.8%) was reported in diarrhoeic calf isolates in another study in Egypt [25]; however, Chen et al. [9] failed to detect any PMQR genes in cattle isolates in China. In contrast to animal isolates, none of the human isolates contained PMQR genes. In China, 8% of human diarrhoeic isolates harboured PMQR genes [9]. 

In this study, CR genes were detected in 14.3% of isolates (three diarrhoeic calves and one worker isolate). Despite the lack of carbapenem use in veterinary practice, an emerging increase in CR genes acquired among *E. coli* isolates from cattle has been reportedworldwide [13,16]. In Egypt, despite very few reports, *E. coli* isolates carrying CR genes were previously reported in faeces of healthy cattle [26], in milk, in faeces of diarrhoeic cattle, and in the stool samples of diarrhoeic humans [13]. *bla*_VIM_ gene was the only detected CR gene in this study. Similarly, this gene was reported in cattle *E. coli* isolates from Egypt [13] and elsewhere [16].

The most detected AMR genotypes in our study were *bla*_SHV_/- (17.9%, five O26 isolates), *bla*_SHV_/*bla*_VIM_ (14.3%, four O26 isolates), *bla*_TEM-1_/*bla*_CTX-M1_ (10.7%, three O111 isolates), and *bla*_TEM_/*bla*_CTX-M-1_/*qnrA*/*qnrS* (7.1%, two O111 isolates). In line with our finding, cattle isolates harbouring one ESBL gene were the predominant genotype in Germany [14]. In contrast, other studies in Egypt showed that cattle isolates with multiple ESBL and/or combined ESBL/PMQR genes were more frequent than isolates with a single ESBL gene [13,25,26]. Interestingly, Elmonir et al. [13] detected O26 isolates harbouring *bla*_VIM_ combined with ESBL genes (ESBL/CR genotype) in milk and diarrhoeic (calves and human) cases in another study in the same research area (Kafrelsheikh governorate). This suggests clonal dissemination and the possible emergence of this CR-O26 serovar in the study area. 

All EPEC isolates in this study (100%) were MDR, with a MAR index ranging from 0.23 to 0.92. This is higher than previous reports (10.4–77.3%) from dairy farms in Egypt [6,25] and elsewhere [5,12]. The detected EPEC showed high resistance rates (71–100%) for AMP, AMC, NA, TE, SXT, and KZ. These findings are in agreement with previous reports worldwide [5,6,12]. 

Around two-thirds (64.3%) of the isolates were resistant to CIP. A similarly high resistance rate (41.3%) was recorded for fluoroquinolones in Turkey [5]; however, a much lower rate (1.5%) was reported in China [20]. The lowest resistance rates were reported for CN (7.1%) and IPM (14.3%). No resistance to CN and IPM was previously reported [6,20]; however, resistance to carbapenems among cattle isolates was reported in previous studies in Egypt [13,26]. There was a concordance between the acquisition of AMR genes and the expression of phenotypic resistance to corresponding antibiotics (β-lactam, carbapenem, and fluoroquinolones) in all EPEC isolates in this study. However, few isolates lacking any of the investigated AMR genes showed phenotypic resistance to β-lactam and/or fluoroquinolones (ID 6, 7, and 20), which may be attributed to other AMR genes or mechanisms that were not studied in this research.

Variance in the rates of AMR genes acquisition/phenotypic resistance in EPEC isolates recovered in this study, as well as previous studies conducted elsewhere, may be linked to various factors, including ecological factors (environmental conditions), host factors (previous antibiotic therapy), genetic factors (virulence or mobile genetic elements), or technical factors (sampling and detection techniques).

REP-PCR-based genotyping analysis of the 28 EPEC isolates proved the high genetic diversity (6 Cs and 27 RTs), regardless of the sample sources, although isolates of the same serotype were clustered together. This is consistent with previous reports in Egypt [13] and elsewhere [27]. Interestingly, isolates of the same serotype that shared the same farm showed higher genetic similarity than isolates of this serotype in another farm (for example, O26 isolates in Farm 2 displayed 85–95% similarity, although they shared only 75% similarity with O26 isolates from Farm 3). This farm (location)-associated genetic similarity was previously reported worldwide [15,28]. These findings prove vertical and horizontal clonal dissemination of EPEC serovars between different sources within the farm, as previously reported [14,15,28]. 

Importantly, O111 and O26 worker isolates (ID 3 and 17, respectively) showed high genetic similarity (80–95%) with matched serotypes of diarrhoeic calves (O111 ID 2 and O26 ID 18, respectively). This may highlight potential interspecies zoonotic transmission of these pathogens between workers and calves within the farm, as reported in other studies [14,23]. This means that dairy farm workers are at constant occupational risk and may also play a role in reverse transmission of MDR *E. coli* isolates and/or their AMR genes within the farm [14,15]. Furthermore, Silvestro et al. [23] provided genetic evidence of infection transmission from the dairy workers to their families; therefore, they may also pose a health risk to their contacts.

## 4. Materials and Methods

### 4.1. Sampling

Samples were collected from three dairy farms in the Kafrelsheikh district of the Kafrelsheikh governorate. The governorate is located in the northern region of the Nile delta of Egypt. We collected a total of 240 samples from diarrhoeic calves (75 calves), dairy cows (150 milk samples), and farm workers (15 workers) on three dairy farms during 2019. We collected rectal swabs samples from diarrhoeic calves (those with ≥3 loose faeces per day) ranging in age from 1 day to 4 months. We collected composite milk samples (150–200 mL per animal) from individual dairy cows that showed no signs of mastitis or ill health. Stool samples were voluntarily provided in sterile containers by individual workers from each farm.

### 4.2. Isolation, Identification, and Serotyping of Enteropathogenic E. coli (EPEC)

We inoculated faecal calf samples and worker stool samples on MacConkey’s agar (Oxoid, Hampshire, UK), and the plates were incubated at 37 ℃ for 24 h. Lactose fermenter (pink in colour) colonies were then subcultured on eosin methylene blue agar (EMB; Oxoid) and were incubated under the same conditions. For milk samples, ten dilutions on Tryptone Soy broth (TSB; Oxoid) were incubated at 37 ℃ for 6 h, and then an inoculum of each sample was cultured on MacConkey’s agar, followed by EMB agar at 37 ℃ for 24 h each. Suspected *E. coli* colonies on EMB (green metallic sheen in colour) were biochemically confirmed by API-20E (bioMérieux, Marcy-l’Etoile, France). All EPEC isolates were serotyped using polyvalent and monovalent O-antisera sets (Denka Seiken Co., Tokyo, Japan) according to the manufacturer’s instructions.

### 4.3. Molecular Confirmation of EPEC 

All *E. coli* isolates were examined for detection of *eae*, *bfpA*, *stx1*, and *stx2* virulence genes. DNA of each isolate was extracted from an overnight TSB culture using a QIAamp DNA Mini Kit (Qiagen, Hilden, Germany) according to the manufacturer’s instructions. Uniplex PCR was conducted for *eae* gene detection. The PCR mix contained 12.5 μL of EmeraldAmp MAX PCR master mix (Takara Bio, Kusatsu, Japan), 1 μL (20 pmol) of each primer, 5 μL of DNA template (~50 ng), and water up to a final volume of 25 μL. The PCR reaction was conducted in an Applied Biosystem 2720 thermal cycler (Applied Biosystem, Foster City, CA, USA) under the following cycling conditions: 94 °C for 7 min; 35 cycles of 94 °C for 1 min, 51 °C for 1 min, and 72 °C for 2 min; and a final extension at 72 °C for 10 min. *bfpA*, *stx1*, and *stx2* genes were detected under the same PCR conditions as for *eae*. The primers and annealing temperature specific to each gene are illustrated in Supplementary Data (Table S1). *E. coli* O157:H7 Sakai (positive for *stx1* and *stx2* genes) and *E. coli* strain E2348/69 (positive for *eae* and *bfpA* genes) were used as positive controls in all PCR reactions. Isolates that were positive for *eae* gene and negative for *stx1* and *stx2* genes were defined as EPEC [1]. EPEC isolates that were negative for *bfpA* gene were considered atypical EPEC (aEPEC). 

### 4.4. Phenotypic Antimicrobial Susceptibility Testing of EPEC Isolates

The Kirby–Bauer disk diffusion technique was used to determine antibiotic sensitivity following the guidelines of the Clinical and Laboratory Standards Institute [29]. Thirteen antibiotic discs (Oxoid, Hampshire, UK) were used: ampicillin (AMP, 10 μg), amoxicillin-clavulanic acid (AMC, 30 μg), cephazolin (KZ, 30 μg), cefotaxime (CTX, 30 μg), cefepime (FEB, 30 μg), aztreonam (ATM, 30 μg), imipenem (IPM, 10 μg), nalidixic acid (NA, 30 μg), ciprofloxacin (CIP, 5 μg), tetracycline (TE, 30 μg), sulfamethoxazole-trimethoprim (SXT, 25 μg), azithromycin (AZM, 15 μg), and gentamicin (CN, 10 μg). A double-disk synergy test and a modified Hodge test were conducted to confirm ESBL and carbapenemase production, respectively [13]. The reference strains *E. coli* NCTC 13353 (positive control for ESBL), *E. coli* ATCC BAA-2469 (positive control for carbapenemase), *Klebsiella pneumoniae* NCTC 13439 (positive control for fluoroquinolone), and *E. coli* ATCC 25922 (negative control) were used for quality control in all conducted tests. The multiple antibiotic resistance (MAR) index was calculated as previously described [13].

### 4.5. Molecular Detection of Antimicrobial Resistance Genes in EPEC Isolates

We screened all EPEC isolates using a uniplex PCR to detect each of the following antibiotic resistance genes: ESBL-encoding genes (*bla*_TEM_, *bla*_CTX-M-1_, *bla*_OXA-1_*,* and *bla*_SHV_), carbapenemase-encoding genes (*bla*_VIM_, *bla*_NDM-1_, and *bla*_IMP_), and PMQR genes (*qnrA*, *qnrB,* and *qnrS*). The PCR mix and cycling conditions were the same as those used for *eae*, with the exception of the primer sets and annealing temperature specific to each gene (Table S1). The following reference strains were used for quality control: *K. pneumoniae* ATCC BAA-1705 (*bla*_TEM_ and *bla*_SHV_), *E. coli* NCTC 13353 (*bla*_CTX-M-1_), *E. coli* NCTC 13476 (*bla*_IMP_), *E. coli* ATCC BAA-2469 (*bla*_NDM-1_), and *K. pneumoniae* NCTC 13439 (*bla*_VIM-1_ and *qnrS*). Additionally, an *E. coli* isolate positive for *qnrA* and *qnrB* was kindly provided by the Central Laboratory of the Faculty of Veterinary Medicine, Kafrelsheikh University, Kafr el-Sheikh, Egypt. The *E. coli* ATCC 25922 reference strain was used as a negative control for all PCR tests.

### 4.6. Sequencing of EPEC Isolates Positive for bla_CTX-M-1_ and bla_SHV_

We selected two O111 isolates positive for *bla*_CTX-M-1_ (ID 2 from diarrhoeic calves and ID 3 from a worker) and three O26 isolates positive for *bla*_SHV_ (ID 13 and 18 from diarrhoeic calves and ID 17 from a worker). These isolates showed the highest MAR indices (0.77–0.92). The PCR products were purified using a QIAquick gel extraction kit (Qiagen, Valencia, CA, USA). PCR product sequencing in both directions was conducted in an Applied Biosystems 3130 genetic analyser (Applied Biosystems, Foster City, CA, USA) using a BigDye Terminator v3.1 cycle sequencing kit (Applied Biosystems) following the manufacturer’s instructions. The nucleotide sequence identity for each gene was confirmed using the BLAST 2.2 program (National Center for Biotechnology Information, NCBI). The accession numbers of the sequenced *bla*_CTX-M-1_ gene are MW721313 (isolate ID 2) and MW721311 (isolate ID 3). The accession numbers of the sequenced *bla*_SHV_ gene are MW721318 (isolate ID 13), MW721319 (isolate ID 18), and MW721320 (isolate ID 17).

### 4.7. Genotyping of EPEC Isolates

We genotyped the EPEC isolates by the REP-PCR method as previously described [30]. We constructed a REP-PCR-based dendrogram using the Dice coefficient and the unweighted pair group method with arithmetic mean in GelJ v.2.0 software [31]. The Simpson’s discrimination index for REP-PCR genotyping was calculated according to Hunter and Gaston [32].

### 4.8. Statistical Analysis

The odds ratio and potential association between antibiotic resistance traits and the source of EPEC isolates were assessed using a univariate logistic regression model. Statistical analyses were conducted using SPSS v19 software (IBM, Armonk, NY, USA), with significance set at *p* ≤ 0.05. 

## 5. Conclusions

In conclusion, this study showed a high rate of MDR-EPEC-carrying AMR genes from various sources (especially diarrhoeic calves) in dairy farms in Egypt. This highlights the potential public health risk of these pathogens or their AMR genes crossing to humans in contact with dairy cattle or milk consumers in Egypt. Restriction of unauthorised use of antibiotics in dairy farms, use of protective clothing, enhanced awareness among workers, and extended surveillance for MDR-EPEC at a national level are mandatory prevention measures. Research is currently underway to further characterize the MDR-EPEC from other hosts in a wide geographical area. 

## Figures and Tables

**Table 1:** Frequency distribution of EPEC and associated AMR traits in samples collected from different sources in dairy farms.

Isolates			Source	Positive (%)	Univariate Regression	
					Odds (CI 95%)	*p*-Value
EPEC 28/240 (11.7)			Milk	8/150 (5.3)	-	-
			Diarrhoea	17/75 (22.7)	5.2 (2.1–12.7)	˂0.001
			Worker	3/15 (20)	4.4 (1.04–18.9)	0.04
AMR Traits	Phenotypic	MDR-EPEC 28/240 (11.7)	Milk	8/150 (5.3)	-	-
			Diarrhoea	17/75 (22.7)	5.2 (2.1–12.7)	˂0.001
			Worker	3/15 (20)	4.4 (1.04–18.9)	0.04
	Genetic	AMR-EPEC 22/240 (9.2)	Milk	6/150 (4)	-	-
			Diarrhoea	14/75 (18.7)	5.5 (2.02–15.01)	0.001
			Worker	2/15 (13.3)	3.7 (0.7–20.2)	0.1
		ESBL-EPEC 21/240 (8.8)	Milk	6/150 (4)	-	-
			Diarrhoea	13/75 (17.3)	5.03 (1.8–13.8)	0.002
			Worker	2/15 (13.3)	3.7 (0.7–20.2)	0.1
		PMQR-EPEC 7/240 (2.9)	Milk	1/150 (0.7)	-	-
			Diarrhoea	6/75 (8)	12.9 (1.5–109.7)	0.02
		CR-EPEC 4/240 (1.7)	Diarrhoea	3/75 (4)	-	-
			Worker	1/15 (6.7)	1.7 (0.2–17.7)	0.7
	AMR Genotypes	ESBL/- 11/240 (4.6)	Milk	5/150 (3.3)	-	-
			Diarrhoea	5/75 (6.7)	2.1 (0.6–7.4)	0.3
			Worker	1/15 (6.7)	2.1 (0.2–18.9)	0.5
		ESBL/PMQR 6/240 (2.5)	Milk	1/150 (0.7)	-	-
			Diarrhoea	5/75 (6.7)	10.6 (1.2–92.8)	0.03
		ESBL/CR 4/240 (1.7)	Diarrhoea	3/75 (4)	-	-
			Worker	1/15 (6.7)	1.7 (0.2–17.7)	0.7

**Table 2:** Frequency distribution of EPEC serovars per source.

Source	Sample No.	O26		O111		O124		O126		O55	
		No.	%	No.	%	No.	%	No.	%	No.	%
Diarrhoeic calves	75	7	9.3	5	6.7	1	1.3	2	2.7	2	2.7
Milk	150	4	2.7	2	1.3	2	1.3	0	0	0	0
Workers	15	1	6.7	1	6.7	1	6.7	0	0	0	0
Total	240	12	5	8	3.3	4	1.7	2	0.8	2	0.8

**Table 3:** Frequency distribution of AMR genotypes per source.

Isolate AMR Genotypes (%)	Cattle		Workers	Total n = 28
	D n = 17	M n = 8	St n = 3	
**I- ESBL/- genotype**	**5 (29.4)**	**5 (62.5)**	**1 (33.3)**	**11 (39.3)**
*bla*_SHV_	2 (11.8)	3 (37.5)	0 (0)	5 (17.9)
*bla*_TEM_	1 (5.9)	1 (12.5)	0 (0)	2 (7.1)
*bla*_CTX-M-1_	1 (5.9)	0 (0)	0 (0)	1 (3.6)
*bla*_TEM-1_, *bla*_CTX-M-1_	1 (5.9)	1 (12.5)	1 (33.3)	3 (10.7)
**II- PMQR/- genotype**	**1 (5.9)**	**0 (0)**	**0 (0)**	**1 (3.6)**
*qnrA*	1 (5.9)	0 (0)	0 (0)	1 (3.6)
**III- ESBL/PMQR genotype**	**5 (29.4)**	**1 (12.5)**	**0 (0)**	**6 (21.4)**
*bla*_TEM_, *bla*_CTX-M-1_, *qnrA*, *qnrS*	2 (11.8)	0 (0)	0 (0)	2 (7.1)
*bla*_TEM_, *bla*_CTX-M-1_, *qnrB*	1 (5.9)	0 (0)	0 (0)	1 (3.6)
*bla*_TEM_, *qnrA*, *qnrB*	2 (11.8)	0 (0)	0 (0)	2 (7.1)
*bla*_TEM_, *qnrA*	0 (0)	1 (12.5)	0 (0)	1 (3.6)
**IV- ESBL/CR genotype**	**3 (17.6)**	**0 (0)**	**1 (33.3)**	**4 (14.3)**
*bla*_SHV_, *bla*_VIM_	3 (17.6)	0 (0)	1 (33.3)	4 (14.3)
**Total**	**14 (82.4)**	**6 (75)**	**2 (66.7)**	**22 (78.6)**

D, diarrhoeic calves; M, milk; St, stool.

## Data Availability

The authors confirm that the data supporting the findings of this study are available within the article.

## Supplementary Materials

The following supporting information can be downloaded at: https://www.mdpi.com/article/10.3390/antibiotics11080999/s1. Figure S1: Sequence alignment of blaCTX-M-1 gene of diarrhoeic calf isolate ID 2 (accession no.: MW721313) and worker isolate ID 3 (accession no.: MW721311) with the blaCTX-M-15 gene of other *E. coli* isolates in GenBank; Figure S2: Sequence alignment of the blaSHV gene of the two diarrhoeic calf isolates (ID 13, MW721318; and ID 18, MW721319) and a worker isolate (ID 17, MW721320) with the blaSHV-12 gene of other E. coli isolates in GenBank; Table S1: Primers and PCR cycling conditions used in this study. References [13,30,33,34,35,36,37] are cited in the supplementary materials.
