# Supplementary material for: Multidrug-Resistant Enteropathogenic Escherichia coli Isolated from Diarrhoeic Calves, Milk, and Workers in Dairy Farms: A Potential Public Health Risk
Source: Antibiotics (Basel). 2022 Jul 25;11(8):999. doi: 10.3390/antibiotics11080999 (PMC9332572; doi:10.3390/antibiotics11080999)
Supplement: Supplementary file 1 [file antibiotics-11-00999-s001.zip › antibiotics-1829382-supplementary.pdf]

Table S1: Primers and PCR cycling conditions of this study

Target gene	Primers sequences (5' – 3')	Product (bp)	Annealing Temp.	Reference
**REP-PCR**	REP1R: IIIICGICGICATCIGGC	Variable	40ºC	**[1]**
	REP2: ICGICTTATCIGGCCTAC			
eaeA	ATGCTTAGTGCTGGTTTAGG	248	51ºC	**[2]**
	GCCTTCAT ATTTCGCTTTC			
bfpA	AATGGTGCTTGCGCTTGCTGC	326	56ºC	**[3]**
	GCCGCTTTATCCAACCTGGTA			
stx1	ACACTGGATGATCTCAGTGG	614	60ºC	**[4]**
	CTGAATCCCCCTCCATTATG			
stx2	CCATGACAACGGACAGCAGTT	779	60ºC	
	CCTGTCAACTGAGCAGCACTTTG			
***bla*$_{\mathbf{TEM}}$**	CATTTCCGTGTCGCCCTTATTC	800	61ºC	
	CGTTCATCCATAGTTGCCTGAC			
***bla*$_{\mathbf{OXA\text{-}1}}$**	GGCACCAGATTCAACTTTCAAG	564	61ºC	
	GACCCCAAGTTTCCTGTAAGTG			
***bla*$_{\mathbf{SHV}}$**	AGGATTGACTGCCTTTTTG	392	54ºC	**[5]**
	ATTTGCTGATTTCGCTCG			
***bla*$_{\mathbf{CTX\text{-}M\text{-}1}}$**	ATGTGCAGYACCAGTAARGTKATGGC	593	54ºC	**[6]**
	TGGGTRAARTARGTSACCAGAAYCAGCGG			
***bla*$_{\mathbf{IMP}}$**	CATGGTTTGGTGGTTCTTGT	488	55ºC	**[4]**
	ATAATTTGGCGGACTTTGGC			
***bla*$_{\mathbf{VIM}}$**	AGTGGTGAGTATCCGACA	280	52ºC	
	ATGAAAGTGCGTGGAGAC			
***bla*$_{\mathbf{NDM\text{-}1}}$**	GGCGGAATGGCTCATCACGA	287	58ºC	
	CGCAACACAGCCTGACTTTC			
qnrA	ATTTCTCACGCCAGGATTTG	516	53ºC	**[7]**
	GATCGGCAAAGGTTAGGTCA			
qnrB	GATCGTGAAAGCCAGAAAGG	469	53ºC	
	ACGATGCCTGGTAGTTGTCC			
qnrS	ACGACATTCGTCAACTGCAA	417	53ºC	
	TAAATTGGCACCCTGTAGGC			

References

1. Mohapatra, B.R.; Broersma, K.; Mazumder, A. Comparison of five rep-PCR genomic fingerprinting methods for differentiation of fecal Escherichia coli from humans, poultry and wild birds. *FEMS microbiology letters* **2007**, *277*, 98-106.
2. Bisi-Johnson, M.A.; Obi, C.L.; Vasaikar, S.D.; Baba, K.A.; Hattori, T. Molecular basis of virulence in clinical isolates of Escherichia coli and Salmonella species from a tertiary hospital in the Eastern Cape, South Africa. *Gut pathogens* **2011**, *3*, 1-8.
3. Gunzburg, S.T.; Tornieporth, N.G.; Riley, L.W. Identification of enteropathogenic Escherichia coli by PCR-based detection of the bundle-forming pilus gene. *Journal of clinical microbiology* **1995**, *33*, 1375-1377.
4. Elmonir, W.; Shalaan, S.; Tahoun, A.; Mahmoud, S.F.; Remela, E.M.A.; Eissa, R.; El-Sharkawy, H.; Shukry, M.; Zahran, R.N. Prevalence, antimicrobial resistance, and genotyping of Shiga toxin-producing Escherichia coli in foods of cattle origin, diarrheic cattle, and diarrheic humans in Egypt. *Gut Pathogens* **2021**, *13*, 1-11.
5. Colom, K.; Pérez, J.; Alonso, R.; Fernández-Aranguiz, A.; Lariño, E.; Cisterna, R. Simple and reliable multiplex PCR assay for detection of bla TEM, bla SHV and bla OXA–1 genes in Enterobacteriaceae. *FEMS microbiology letters* **2003**, *223*, 147-151.
6. Archambault, M.; Petrov, P.; Hendriksen, R.S.; Asseva, G.; Bangtrakulnonth, A.; Hasman, H.; Aarestrup, F.M. Molecular characterization and occurrence of extended-spectrum β-lactamase resistance genes among Salmonella enterica serovar Corvallis from Thailand, Bulgaria, and Denmark. *Microbial Drug Resistance* **2006**, *12*, 192-198.
7. Robicsek, A.; Strahilevitz, J.; Sahm, D.; Jacoby, G.; Hooper, D. qnr prevalence in ceftazidime-resistant Enterobacteriaceae isolates from the United States. *Antimicrobial agents and chemotherapy* **2006**, *50*, 2872-2874.

Supplementary Figures

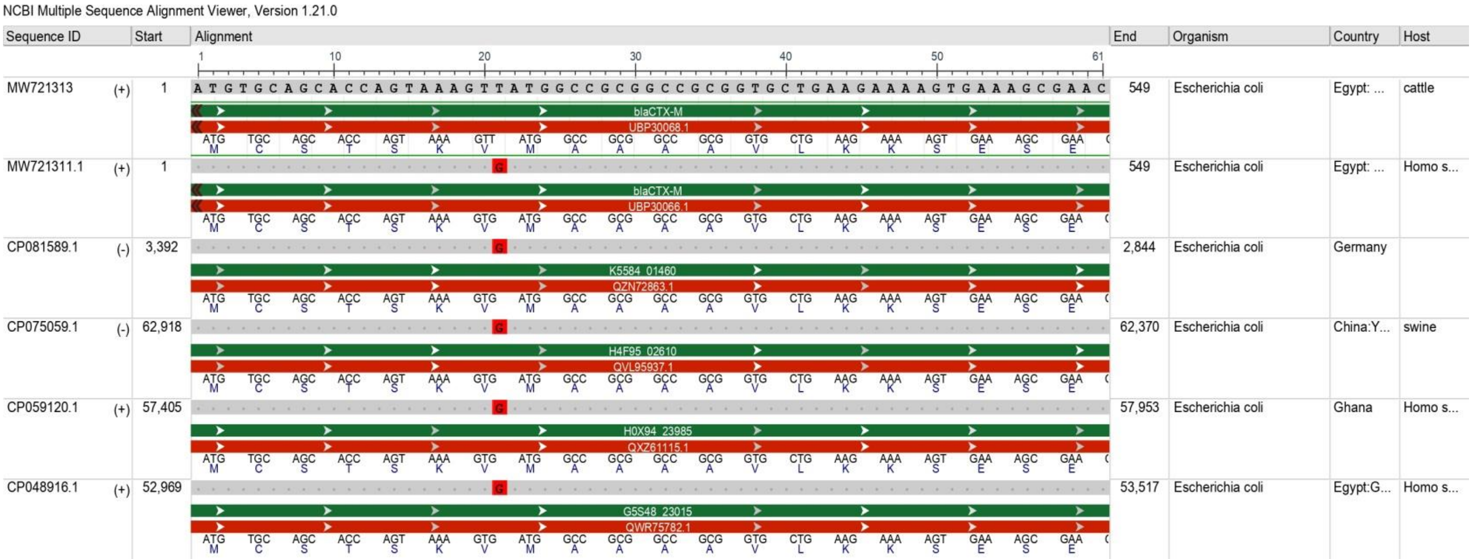

Figure S1. Sequence alignment of *bla*$_{CTX-M-1}$ gene of diarrheic calf isolate ID 2 (accession no.: MW721313) and worker isolate ID 3 (accession no.: MW721311) with *bla*$_{CTX-M-15}$ gene of other *E. coli* isolates on GenBank.

* Red shadow: single nucleotide mutation (G to T at position 21).

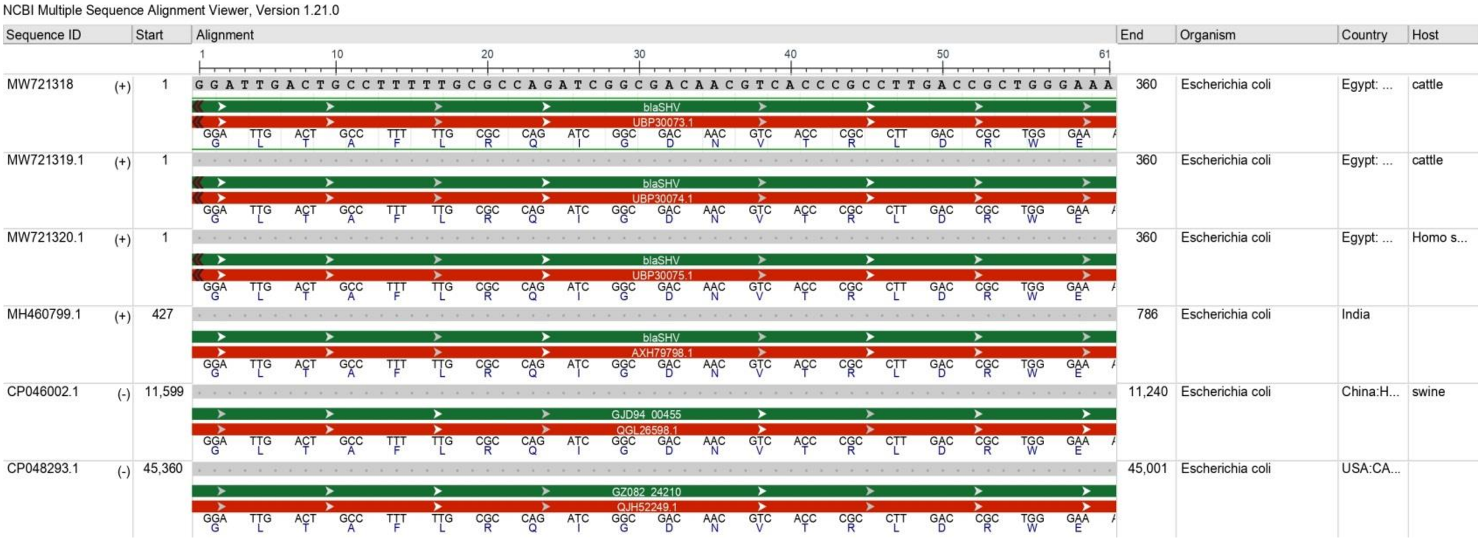

Figure S2. Sequence alignment of *bla*$_{SHV}$ gene of the two diarrheic claves isolates (ID 13, MW721318 and ID 18, MW721319), and a worker isolate (ID 17, MW721320) with *bla*$_{SHV-12}$ gene of other *E. coli* isolates on GenBank.
